# Infectious hematogenous lumbar spondylodiscitis caused by *Actinotignum schaalii* in a 74-year-old man: A case report

**DOI:** 10.1016/j.ijscr.2022.107453

**Published:** 2022-07-25

**Authors:** Ekkehard F. Röpke, Martin Chwoika, Tim Treber, Jens Meyer, Christoph Paasch

**Affiliations:** a Department of Orthopaedics, Traumatology and Spine Surgery, Helios Klinik Jerichower Land, Burg, Germany; b Medical Care Center for Laboratory Medicine, Microbiology, Hygiene and Human Genetics “Prof. Schenk/Dr. Ansorge & Kollegen”, Magdeburg, Germany; c Clinic for General and Visceral Surgery, Klinikum Magdeburg gGmbH, Germany.; d University Hospital Brandenburg an der Havel, Brandenburg Medical University, Germany; e Brandenburg Medical School, Faculty of Medicine/Faculty of Health Sciences, Germany

**Keywords:** Back pain, Spondylodiscitis, *Actinotignum schaalii*, Immobilising pain, *Actinobaculum schaalii*, Case report, Parkinson's disease

## Abstract

• Haematogenous bacterial spondylodiscitis due to infection with a well-treatable but easily overlooked and often underdiagnosed pathogen.
• An argument against short pedicle screw instrumentation, when urgent surgical therapy of the infected spine is required, in patients with PD and poor general condition.
• Especially in cases where a bacterial infection is suspected clinically and by imaging, the detection of germs must be forced by all means in order to be able to treat the patients well.

## Abstract (de)

**Introduction and importance:**

Infectious hematogenous spondylodiscitis (HS) requires urgent treatment. In some cases, the germ detection of very slow growing bacteria can be challenging and a life threatening HS can easily be overlooked.

**Presentation of case:**

A Parkinson's patient presented with slowly increasing immobilising back pain for three months and a deterioration of his general condition. A lumbar spondylodiscitis (L1/2) was diagnosed. We indicated surgical exploration with debridement and dorsal instrumented stabilization (L1-2). On the fifth postoperative day, the patient complained again of severe back pain, caused by implant dislocation. We extended the instrumentation in a revision procedure (Th12-L4). After 5 days of incubation, a pan sensitive *Actinotignum schaalii* could be detected microbiologically. The patient was discharged free of infection within 14 days after surgery with appropriate oral antibiotic therapy.

**Clinical discussion:**

*Actinotignum schaalii* is difficult to detect but can be successfully treated. *Actinotignum species* rarely grow under normal urine culture conditions. In chronic inflammation, as a cause of gradual weight loss, anemia and back pain, *Actinotignum schaalii* should be considered as a causative agent in the ageing population. In cases of severe HS and comorbidities, a definitive surgical solution is required.

**Conclusion:**

A HS caused by *Actinotignum schaalii* is rare. Particularly in cases where a bacterial infection is suspected, prolonged incubation under appropriate conditions is essential. Surgical debridement and prolonged antibiotic treatment are substantial to cure the patient.

## 1 Introduction

Spondylodiscitis is an infectious disease of the spine, which requires urgent treatment. The aetiology and pathogenesis can be classified as iatrogenic (postoperative/postinterventional), per continuitatem and haematogenous. Haematogenous spondylodiscitis (HS) can be caused by a broad spectrum of pathogens. The most common bacteria are *Staphylococcus aureus*, followed by Gram-negative rods, coagulase-negative *Staphylococci*, *Streptococci*, *Enterococci*, and anaerobes [1].

The HS includes a variety of infectious localisations within the spine, with discitis, vertebral osteomyelitis and spondylodiscitis. Paravertebral abscesses and spinal empyema might result in different clinical symptoms. Intravenous drug use, severe concomitant medical conditions such as diabetes and renal failure with dialysis treatment, advanced age and immunodeficiency are known risk factors [2]. The incidence of HS has increased in recent years [3]. Advances in medical diagnostic and medical care that extend the life expectancy of elderly and immunocompromised populations, as well as an increase in the number of hospital-acquired infections, may be reasons for this trend [2].

The diagnostic approach consists of magnet resonance imaging, laboratory testing and repeated blood culture analysis [3]. In case of a haematogenous infection of the spine, an infection focus search must be performed to reveal the cause of the infection. Exclusion of heart valve colonization must be considered and germ detection should be enforced. Inflamed tissue can be obtained for diagnosis by CT guided fine needle biopsy [4]. Depending on the severity of the infection, there are conservative treatment options with antibiotics, pain medication and immobilization as well as surgical treatment [3]. If HS remains untreated, it may lead to sepsis, permanent organ damage and death.

We herein present a rare case of severe HS due to infection with *Actinotignum schaalii* in a 74-year-old man.

This pathogen belongs to *t*he genus *Actinotignum*, which contains three species: *Actinotignum urinale*, *Actinotignum sanguinis* and *Actinotignum schaalii* (formerly *Actinobaculum schaalii*) [5], [6]. *Actinotignum schaalii* is a Gram-positive coccoid rod that is part of the urinary microbiota. This species has been described as being responsible for mostly urinary tract infections and bacteraemia in elderly [5], [7]. Pedersen et al. described an incidence of 11 to 1.000.000 per year for *Actinotignum* bacteremia in their retrospective observational study [7]. *Actinotignum schaalii* rarely grows under normal urine culture conditions. For identification a PCR-Test or matrix-assisted laser desorption/ionization time-of-flight mass spectrometry (MALDI-TOF MS) is often needed [6]. *Actinotignum schaalii* can also be found after 2–3 days of incubation in CO_2_ [5], [6]. This bacterium is resistant to trimethoprim and ciprofloxacin, but susceptible to β-lactams [7].

For literature research the first and senior authors independently reviewed the literature using PubMed and Google Scholar. The following search terms were used: “SPONDYLODISCITIS” AND “*ACTINOBACULUM SCHAALII*” AND “ACTINOTIGNUM SCHAALII” AND “BACK PAIN”.

This work has been reported in line with the SCARE 2020 criteria [8].

## 2 Presentation of case

A Caucasian male patient, 74 years of age, was referred by another hospital. For about 3 months, he suffered from weight loss, anemia and increasing immobilising back pain. An underlying malignant disease was ruled out previously by computed tomography of the abdomen, gastroscopy and colonoscopy before referral to our clinic. The medical history includes a Parkinson's disease (PD), reflux esophagitis Los Angeles Type B with Barrett's esophagus, arterial hypertonia, atrial fibrillation and gout. Oral medication consisted of Phenprocoumon, Allopurinol, Levodopa/Carbodopa, Propanolol, and Oxycodone with an extended release 20 mg/d and 10 mg of Oxycodone as needed. Our patient has undergone basal cell carcinoma excision in the past and his wife reported that he had a depressive episode 6 month ago.

During the clinical examination he was afebrile with normal vital signs. Our patient stated a back pain of 8 according to the numeric analog scale. The patient suffered from tingling paresthesias of the thighs and load depending weakness of the lower limbs. Single radiculopathy, menigism or cauda equina and conus medullaris syndromes were clinically excluded. Due to Parkinson's disease, clinical neurological investigations were limited.

Laboratory test revealed elevated concentrations of the C-reactive protein (231 mg/l) with normal leucocytes count (6.9 gpt/l), normal procalcitonin <0.5 μg/l, hypokalemia (3.9 mmol/l) and hemoglobin 6.2 mmol/l.

Sepsis criteria were not met.

**Fig. 1:**
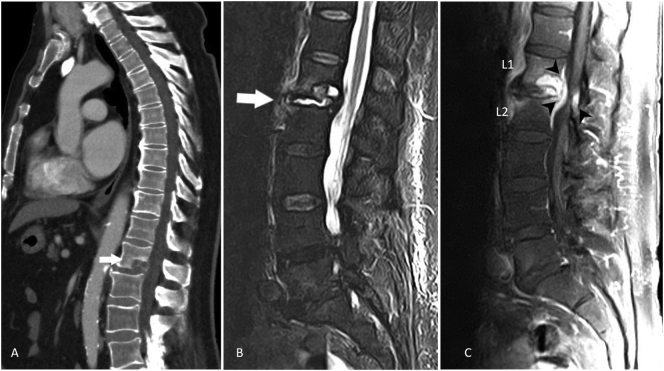
A: A sagittal computed tomography (CT) scan of the thoracolumbar spine shows a suspect lytic lesion in the dorsal region of the first lumbar vertebra (white arrow pointing towards L1). B: preoperative sagittal STIR T2 weighted magnetic resonance imaging (MRI) showing a hyperintense fluid signal within the disc at L1 and L2 (white arrow). C: MRI (T1, sagittal, post contrast medium) shows a significant contrast enhancement of the affected area, with a posterior extension, narrowing the spinal canal (black arrowheads).

The first two blood cultures and the urine analysis at the referring hospital showed no growth of pathogens. The performed CT scan did not reveal any clear infectious pathological findings. Secondary changes with erosion of the first lumbar vertebral body in the context of a possible inflammatory genesis were assumed (Fig. 1).

For further diagnostic and under the suspicion of a spondylodiscitis, a contrast medium enhanced magnetic resonance imaging (MRI) took place. A severe lumbar haematogenous spondylodiscitis (L1/2) was diagnosed. The spinal canal appeared narrowed with a paravertebral abscess formation (Fig. 1). After echocardiographic exclusion of bacterial valve colonization, we indicated surgical treatment of the abscessing spondylodiscitis. Intraoperatively, there was marked spondylodiscitis with inflammatory deposits but without serous purulent exudate. Laminotomy, decompression and surgical debridement of the spinal canal and disc space with dorso-ventral pedicle screw instrumentation L1-L2 was performed (Depuy Synthes Expedium 5.5 Titanium, DePuy Synthes Spine, 700 Orthopaedic Drive Warsaw, IN 46582, USA). A titanium cage as disc replacement was inserted (SPINEART Juliet OL, transforaminal straight cage, SPINEART SA, Chemin du Pré-Fleuri 3, 1228 Plan-Les-Oates, Switzerland). We initiated calculated intravenous antibiotic therapy (4 × 3,5 g Ampicillin/Sulbactam).

**Fig. 2:**
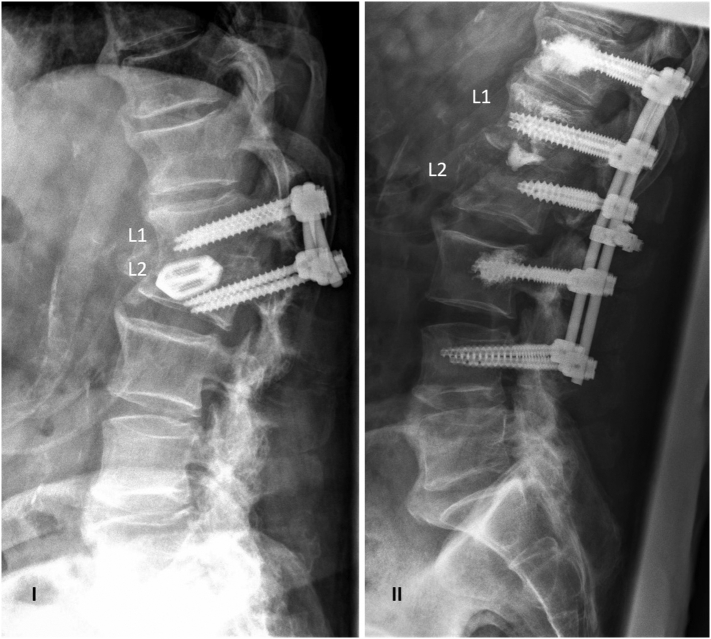
I. The upright lateral thoracolumbar radiography shows the construct failure with displacement of the pedicle screws and intervertebral cage, resulting in a loss of spinal alignment with segmental kyphosis L1/L2. II. Upright lateral thoracolumbar radiography after surgical revision with extended dorsal cement-augmented instrumentation and restoration of spinal alignment.

An increase in the laboratory parameters of infection was seen on the third postoperative day. Microbiological analysis of the intraoperatively resected tissue showed primarily no bacterial growth. After escalation of the intravenous antibiotic therapy (3 × 4,5 g Piperacillin/Tazobactam), infection parameters dropped. On the fifth postoperative day, our patient complained again of severe back pain. Dislocation of the dorsal instrumented stabilization with complete failure of the construct and consecutive kyphosis was detected by radiography (Fig. 2). We removed and exchanged the implants in a revision procedure to a more stable posterior cement-augmented pedicle screw instrumentation Th12-L4 (Depuy Synthes Expedium Viper 2 CFX) (Fig. 2).

**Fig. 3:**
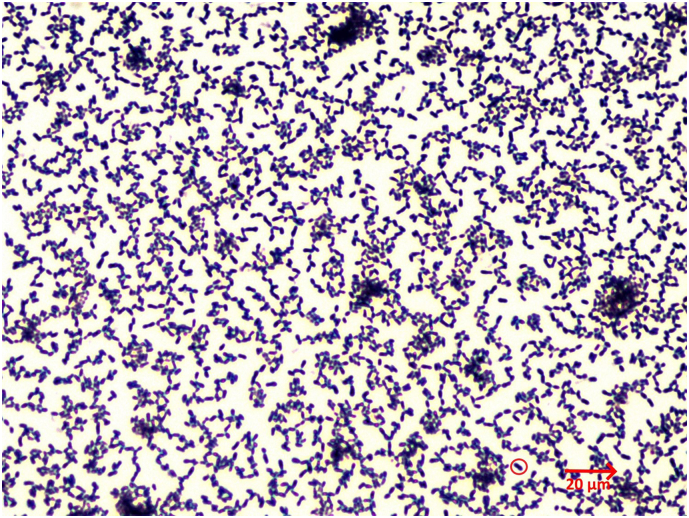
Microscopic image (Gram stain, 1000× magnification) showing Gram-positive coccoid rods (*Actinotignum schaalii*, one specimen in the red circle). (For interpretation of the references to colour in this figure legend, the reader is referred to the web version of this article.)

On day five of incubation, a pan sensitive *Actinotignum schaalii* could be detected using with MALDI-TOF MS (Fig. 3).

The postoperative course was then almost uneventful. The patient could be discharged on day 14 after revision surgery, having survived a clinically mild hospital-acquired SARS-CoV-2 infection. A 3-month course of antibiotic therapy was prescribed (Amoxicillin 3 × 1 g + Rifampicin 2 × 300 mg per os). To date, 12 weeks after the operation, our patient has recovered from the operation within the framework and is living independently in his home environment. He has followed our recommendations for antibiotic therapy. Laboratory results are within normal range and he feels clinically well.

## 3 Discussion

We diagnosed a spondylodiscitis due to an infection with the facultative anaerobic bacterium *Actinotignum schaalii*. This case report adds to one other report of *Actinotignum schaalii* causing spondylodiscitis [9].

Relatively rare pathogens may also cause an HS. Exemplary, Brooks et al. (2022) diagnosed and treated an elderly man with a thoracic HS following urinary sepsis. The authors detected *Klebsiella oxytoca* [10]. In addition, Hsu et al. (2022) operated on a *Salmonella* spondylodiscitis with an epidural abscess [11]. In literature also cases of an HS caused by *Listeria monocytogenes*, *Burkholderia cepacia* and *Mycobacterium chelonae* have been reported [1], [11], [12].

To our knowledge, Haller et al. reported the only clinical case of vertebral osteomyelitis with *Actinotignum schaalii* in 2007. They pointed out the difficulties of diagnosis and the resulting under-representation of the pathogen as the cause of vertebral osteomyelitis. In their case the pathogen was successfully detected with CT-guided biopsy L3/4 and blood culturing. Using 16 s rDNA sequencing, they identified *Actinotignum schaalii*. After eight weeks of iv. antibiotics and mobilisation with a soft-brace, healing was achieved [9]. An HS due to an infection with *Actinotignum schaalii* is rare [7]. It can be speculated that latent infection of low-virulence anaerobic bacteria may play a role in chronic degenerative disc disease because they do not lead to manifestation of clinical infection in immunocompetent patients [13], [14].

It is well known that patients suffering from Parkinson's disease, following thoracolumbar spine fusion surgery have more postoperative complications. Regarding to that, Puvanesarajah et al. [15] published a database analysis on that topic. Individuals with a previous diagnosis of Parkinson's disease (*n* = 4816) and without (*n* = 280,702) were compared. Their multivariate analysis demonstrated that Parkinson's disease was significantly associated with an increased risk for medical complications following thoracolumbar spine fusion surgery [15].

In this case, due to the poor general health condition of our patient, his Parkinson's disease and neurological symptomatic HS, we recommended a short segment fusion surgery to minimise trauma [16], [17]. In retrospect, we have to admit, that the patient was exposed to an overall increased trauma due to the necessary second operation. In our opinion, our case report seems to be an argument for operating patients with poor general condition and Parkinson's disease with extended dorsal pedicle screw instrumentation in case of urgent surgical therapy.

Individuals with Parkinson's disease typically suffer from dystonic tension of the trunk muscles up to camptocormy, which seems to overuse short dorsal instrumentation [18]. Loss of bone quality due to infection may further increase the risk of implant failure.

Due to a lack of scientific reports, no recommendation on a proper therapy is existing. We successfully treated our patient with surgical debridement, dorsal pedicle screw instrumentation, and a prolonged antibiotic therapy.

## 4 Conclusion

An infectious hematogenous spondylodiscitis caused by *Actinotignum schaalii* is extremely rare. Especially in cases where a bacterial infection is suspected and microbiological detection is initially unsuccessful, the detection must be strengthened with all diagnostic possibilities to identify *Actinotignum schaalii*. In presence of neurological symptoms, definitive surgical treatment and prolonged antibiotic therapy might be essential to cure the elderly and morbide.

## Provenance and peer review

Not commissioned, externally peer-reviewed.

## Sources of funding

We acknowledge funding by the MHB Open Access Publication Fund supported by the 10.13039/501100001659German Research Association (DFG).

## Ethical approval

This study was not applicable for ethical approval.

## Consent

Informed consent was obtained from the patient for publication of this case report and accompanying images. A copy of the written consent is available for review by the Editor-in-Chief of this journal on request.

## Author contribution

Dr. med. Ekkehard F. Röpke: Submitting author, examination and surgical therapy, writing, reviewing and editing of the paper.

Dr. med. Martin Chwoika: Microbiological detection of the pathogen.

Tim Treber: Data collection, treatment and examination of the patient.

Jens Meyer: Reviewing and editing.

PD Dr. med. Christoph Paasch: Corresponding author, data analysis and interpretation, supervising writing of the paper.

## Guarantor

Dr. med. Ekkehard Friedrich Röpke.

PD Dr. med. Christoph Paasch.

## Research registration

The case report at hand is not a first-in-man case report of a novel technology or surgical technique, therefore a registration of these case reports according to Declaration of Helsinki 2013 is not required.

## Declaration of competing interest

None.
